# A revised SNP-based barcoding scheme for typing *Mycobacterium tuberculosis* complex isolates

**DOI:** 10.1128/msphere.00169-23

**Published:** 2023-06-14

**Authors:** Egor Shitikov, Dmitry Bespiatykh

**Affiliations:** 1 Department of biomedicine and genomics, Lopukhin Federal Research and Clinical Center of Physical-Chemical Medicine of Federal Medical Biological Agency, Moscow, Russia; Nanjing University of Chinese Medicine, Nanjing, Jiangsu, China

**Keywords:** tuberculosis, barcoding, *Mycobacterium tuberculosis* complex, SNPs, genotyping

## Abstract

The development of whole-genome sequencing technologies is gradually leading to a more detailed description of the population structure of the *Mycobacterium tuberculosis* complex (MTBC). In this study, we correlated previously published classifications on a collection of more than 10,000 genomes and proposed a new, comprehensive nomenclature that unifies the existing ones. In total, we identified 169 lineages and sublineages of *M. tuberculosis*/*M. africanum* and 9 animal-adapted species. For the purpose of organizing these genotypes in a more streamlined manner, we stratified them into five hierarchical levels. To represent the classification and compare it with the reference, we compiled a confirmatory data set of 670 high-quality isolates, which includes all genotypes and species of MTBC, and this confirmatory data set can serve as a basis for further studies. We proposed a set of 213 robust barcoding single-nucleotide polymorphisms and a suitable workflow for reliable differentiation of genotypes and species within the complex. This work integrates the results of all the major systematized studies to date to provide an understanding of the global diversity of the MTBC population structure. The results of this work may ultimately help to reliably determine the pathogen genotype and associate it with traits that reflect its prevalence, virulence, vaccination, and treatment efficiency, as well as to reliably find natural features revealed during its spread.

**IMPORTANCE:**

Through years of research into the *Mycobacterium tuberculosis* complex (MTBC), a number of ambiguous phylogenetic classifications have emerged, which often overlap with one another. In the present study, we have combined all major studies on MTBC classification and inferred a unified, most complete to date classification and accompanying SNP barcodes.

## OBSERVATION

The cause of tuberculosis, the MTBC, remains a pressing issue for both public health and scientists around the world. To date, nine human-adapted lineages and nine animal-adapted species have been identified within the MTBC (1,2). Several techniques have been developed for the MTBC genotyping over the years of research. However, only large sequence polymorphisms (LSPs) in regions of difference and single-nucleotide polymorphisms (SNPs) are the least susceptible to the effects of homoplasia and are the most suitable for phylogenetic purposes (3,4).

A landmark study on phylogenetic polymorphisms was conducted by Coll et al. in 2014 (4), and this classification was updated by Napier et al. in 2020 (1). The classification reflects the hierarchical relationship between lineages and sublineages. In order to distinguish the main phylogenetic lineages and species within the MTBC, a set of 90 validated SNPs was proposed by the authors, wherein the numbering of the main lineages corresponded to the Gagneux et al. classification published in 2006 (5), which was based on LSPs. The latter is a disadvantage of the classification, since it did not take into account additional findings of those years based on other phylogenetic markers. The limitations and shortcomings of the aforementioned classification have led to the emergence of additional barcoding and naming schemes, which often overlap with one another (6).

In order to establish a comprehensive and unified classification system for the various lineages and species within the MTBC, we conducted a rigorous cross-referencing of published studies, utilizing SNP-based genotyping. Our analysis resulted in a comprehensive MTBC classification that effectively encompasses all pertinent lineages and species. In brief, five studies were analyzed, using the Napier et al. (1) typing scheme and corresponding barcodes as the primary reference point. The discovered genotypes were validated on the exploratory data set comprising >10,000 sequenced MTBC isolates obtained from the NCBI sequence read archive (https://www.ncbi.nlm.nih.gov/sra). To construct the most complete phylogenetic structure of the MTBC, a subset of 670 isolates was selected to represent five isolates per each genotype and animal-adapted species (if the number of isolates for a specific genotype/species was not sufficient, the rule was omitted). A system of SNP barcodes, a workflow for reliable MTBC genotyping, and a companion web app were created at the final stage (see Text S1 for comprehensive analysis details).

In total, from the previously described genotypes, we have identified 169 lineages and sublineages of *M. tuberculosis*/*M. africanum* and 9 animal-adapted species. In the resulting classification, we used a five-level system, where the first level corresponds to the main phylogenetic lineage/species, and the last level corresponds to the final phylogroup.

**Fig 1:**
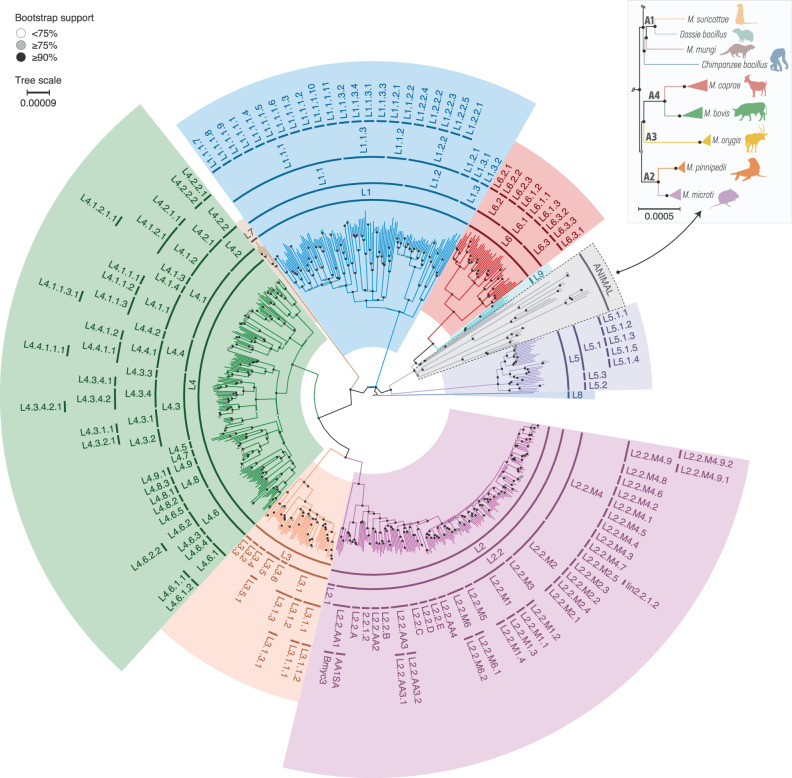
Whole-genome phylogeny of 670 isolates spanning all MTBC genotypes. Maximum-likelihood phylogenetic tree constructed using 42,724 high-quality genome-wide SNPs from 670 MTBC isolates and rooted on *M. canettii* (acc. no. ERR266109 [branch is omitted]), with isolates color coded by main lineage. Bootstrap support values are shown as white <75%, gray ≥75%, or black ≥90% dots on interior nodes.

To represent a unified typing scheme, an aforementioned subset of the MTBC isolates (*n* = 670) was used, including all phylogenetic units: lineage 1 (*n* = 125), lineage 2 (*n* = 180), lineage 3 (*n* = 55), lineage 4 (*n* = 190), lineage 5 (*n* = 35), lineage 6 (*n* = 45), lineage 7 (*n* = 5), lineage 8 (*n* = 1), lineage 9 (*n* = 5), *M. microti* (*n* = 5), *M. pinnipedii* (*n* = 5), *M. orygis* (*n* = 5), *M. bovis* (*n* = 5), *M. caprae* (*n* = 5), *M. suricattae* (*n* = 1), *M. mungi* (*n* = 1), *Dassie bacillus* (*n* = 1), and *Chimpanzee bacillus* (*n* = 1) (Table S1). From the concatenated alignment of 42,724 high-quality genome-wide polymorphic nucleotide sites, a phylogenetic tree was inferred, which showed a structure consistent with previously published phylogenies (Fig. 1) (1,2). Furthermore, for each main lineage, a phylogenetic tree was inferred depicting a correlation with the primary Napier et al. classification (Figs. S1 through S5). The main differences between the two classifications have been described as well (Table S2).

Lineage 2 classification is the most revised one. In 2021, Thawornwattana et al. (7) presented the most comprehensive classification of lineage 2 based on the analysis of over 4,000 isolates from 34 countries. Four additional genotypes from other studies were added to the revised lineage 2 classification, in addition to the aforementioned classification. Lineage 1 and lineage 3 have also undergone extensive revision, at least three major works can be distinguished to complement the classifications of these lineages (8 - 10). In the Napier et al. study (1), the classification for lineage 5 and lineage 6 was only presented by the main lineage but was updated by introducing differentiation into sublineages in a recent work by Coscolla et al. (11). The classification remained the same as the primary one for lineage 4, for which no additional studies were published, as well as for lineage 7, lineage 8, and lineage 9, for which the number of genomes in the public databases is still quite limited. In the case of animal-adapted species, the classification was expanded to include six species that are not listed in the Napier et al. classification (1). It is noteworthy that the phylogenetic analysis of animal-adapted species exhibited similarities with previously published studies, and as a result, they were classified into four distinct clades, namely A1 through A4 (Fig. 1) (2).

Using previously published SNP schemes, we compiled a set of 213 barcoding SNPs (Table S3) for reliable differentiation of 169 *M*. *tuberculosis*/*M. africanum* genotypes and 5 animal-adapted species (*M. mungi*, *M. surricattae*, *D. bacillus,* and *C. bacillus* are represented by single isolates; therefore, they were not used for barcoding). Two SNPs were chosen for each genotype at the first and second levels for better reliability and false-positive exclusion. We verified these 213 barcodes using the confirmatory data set of 670 isolates. All genotypes were correctly called using the proposed barcodes (Table S1). The proposed typing scheme is available and implemented in the reproducible workflow TBvar.

We have developed a web application called TB-gen (https://tb-gen.streamlit.app/) using Streamlit (https://streamlit.io/) to improve the representation of our study results. TB-gen provides a graphical interface to explore our findings. The application includes a curated set of barcoding SNPs and a reference data set with detailed information about isolates. Additionally, phylogenies that visualize the relationships between these isolates are available within the application. Users can also barcode MTBC lineage from the variant call format (VCF) file via the “Genotype lineage” page.

Additionally, we have developed a Python-based command-line tool TbLG (https://github.com/dbespiatykh/tblg), which facilitates the classification of MTBC lineages from a VCF file. The tool employs a curated panel of reference barcoding SNPs, identified in this study, for precise lineage classification.

Taken together, we have introduced the most extensive and flexible level-based MTBC classification scheme, comprising 169 genotypes and 5 animal-adapted species. From this analysis, we identified 213 robust barcoding SNPs and created a workflow for reliable MTBC genotyping. Furthermore, we report the confirmatory data set of 670 MTBC isolates available in public databases, which can serve as a basis for further phylogenetic studies. Overall, our findings might help to more reliably associate the genotype of isolates with phylogeography and such traits of individual genotypes as the incidence of drug resistance, transmission, virulence, vaccination efficiency, and disease severity.

## Data Availability

Reproducible variant calling and lineage barcoding workflow TBvar is available at GitHub (https://github.com/dbespiatykh/TBvar). TB-gen can be accessed at Streamlit cloud (https://tb-gen.streamlit.app) and at GitHub (https://github.com/dbespiatykh/TB-gen). TbLG is available at GitHub (https://github.com/dbespiatykh/tblg) and PyPI (https://pypi.org/project/tblg/).

## SUPPLEMENTAL MATERIAL

Fig S1 msphere.00169-23-s0001.pdf Maximum-likelihood phylogeny of 125 *M. tuberculosis* lineage 1 isolates. Tree was constructed using 15 255 genome-wide SNPs and rooted on *M. tuberculosis* H37Rv (SRR11823427 [branch length is omitted]). The exterior vertical bars and names indicate lineage (black - this study, golden - Napier et al.). Lineages are highlighted with background colors. Bootstrap support values are shown as white <75%, grey ≥75% or black ≥90% dots on interior nodes.

Fig S2 msphere.00169-23-s0002.pdf Maximum-likelihood phylogeny of 180 *M. tuberculosis* lineage 2 isolates. Tree was constructed using 7 919 genome-wide SNPs and rooted on *M. tuberculosis* H37Rv (SRR11823427 [branch length is omitted]). The exterior vertical bars and names indicate lineage (black - this study, golden - Napier et al.). Lineages are highlighted with background colors. Bootstrap support values are shown as white <75%, grey ≥75% or black ≥90% dots on interior nodes.

Fig S3 msphere.00169-23-s0003.pdf Maximum-likelihood phylogeny of 55 *M. tuberculosis* lineage 3 isolates. Tree was constructed using 6 047 genome-wide SNPs and rooted on *M. tuberculosis* H37Rv (SRR11823427 [branch length is omitted]). The exterior vertical bars and names indicate lineage (black - this study, golden - Napier et al.). Lineages are highlighted with background colors. Bootstrap support values are shown as white <75%, grey ≥75% or black ≥90% dots on interior nodes.

Fig S4 msphere.00169-23-s0004.pdf Maximum-likelihood phylogeny of 190 *M. tuberculosis* lineage 4 isolates. Tree was constructed using 21 471 genome-wide SNPs and rooted on *M. canettii* (ERR266109 [branch length is omitted]). The exterior vertical bars and names indicate lineage. Lineages are highlighted with background colors. Bootstrap support values are shown as white <75%, grey ≥75% or black ≥90% dots on interior nodes.

Fig S5 msphere.00169-23-s0005.pdf Maximum-likelihood phylogeny of 120 *M. tuberculosis,* /*M. africanum* isolates from lineage 5, lineage 6, lineage 7, lineage 8, lineage 9 and animal-adapted species. Tree was constructed using 28 520 genome-wide SNPs and rooted on *M. canettii* (ERR266109 [branch length is omitted]). The exterior vertical bars and names indicate lineage. Lineages are highlighted with background colors. Bootstrap support values are shown as white <75%, grey ≥75%, or black ≥90% dots on interior nodes.

Text S1 msphere.00169-23-s0006.docx Study design; materials and methods.

Table S1 msphere.00169-23-s0007.xlsx Confirmatory data set of 670 MTBC isolates.

Table S2 msphere.00169-23-s0008.xlsx Main differences between the two classifications.

Table S3 msphere.00169-23-s0009.xlsx Barcoding SNPs and corresponding studies.

ASM does not own the copyrights to Supplemental Material that may be linked to, or accessed through, an article. The authors have granted ASM a non-exclusive, world-wide license to publish the Supplemental Material files. Please contact the corresponding author directly for reuse.
