# Supplementary figures and images for: A revised SNP-based barcoding scheme for typing Mycobacterium tuberculosis complex isolates
Source: mSphere. 2023 Jun 14;8(4):e00169-23. doi: 10.1128/msphere.00169-23 (PMC10449489; doi:10.1128/msphere.00169-23)

Bootstrap support

○ <75%

● ≥75%

● ≥90%

Tree scale

1e-04

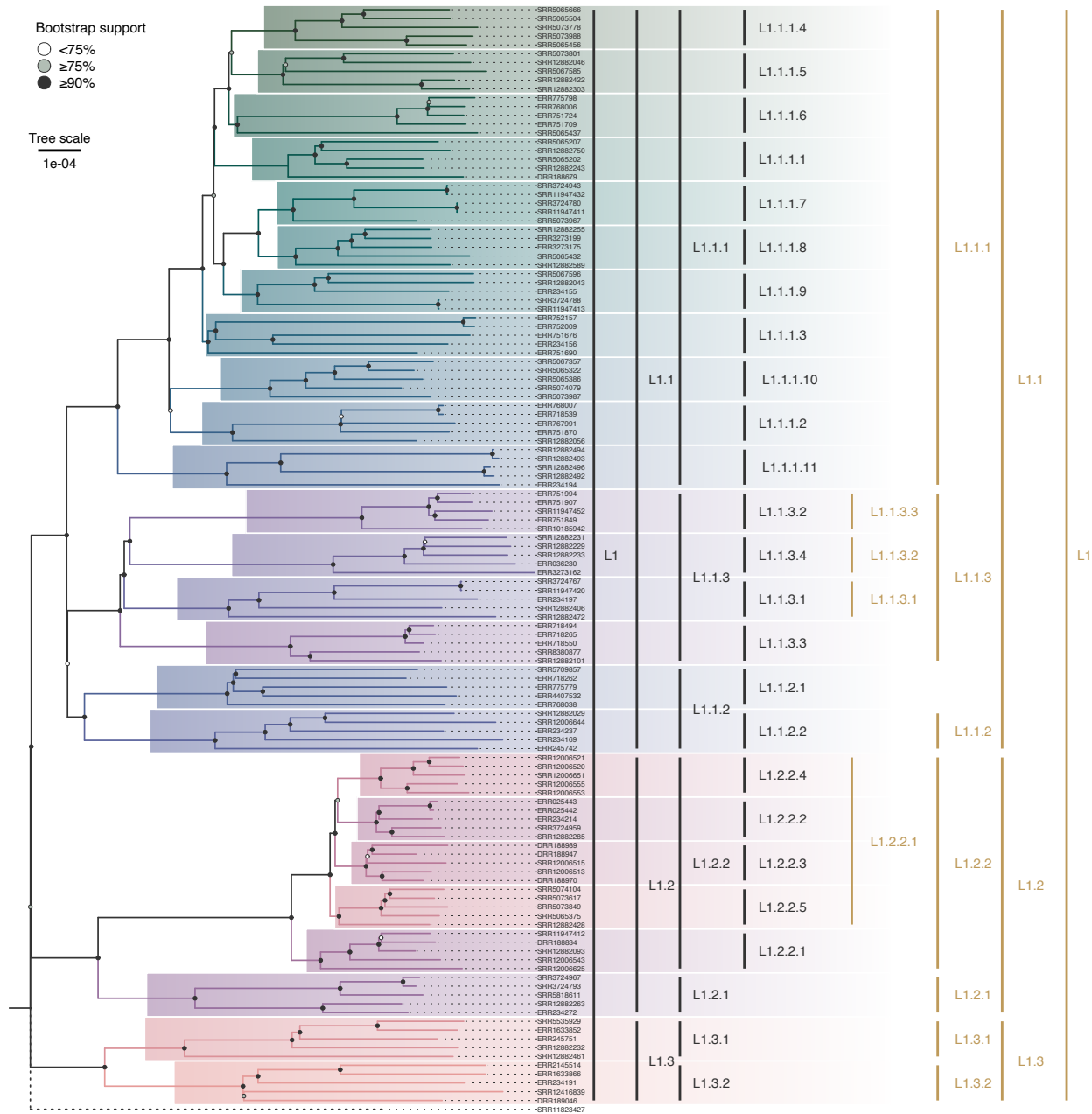

Supplement: Fig S1 — Maximum-likelihood phylogeny of 125 M. tuberculosis lineage 1 isolates. Tree was constructed using 15 255 genome-wide SNPs and rooted on M. tuberculosis H37Rv (SRR11823427 [branch length is omitted]). The exterior vertical bars and names indicate lineage (black - this study, golden - Napier et al.). Lineages are highlighted with background colors. Bootstrap support values are shown as white <75%, grey ≥75% or black ≥90% dots on interior nodes. [file msphere.00169-23-s0001.pdf]

# Bootstrap support

- <75%
- ≥75%
- ≥90%

Tree scale

2e-04

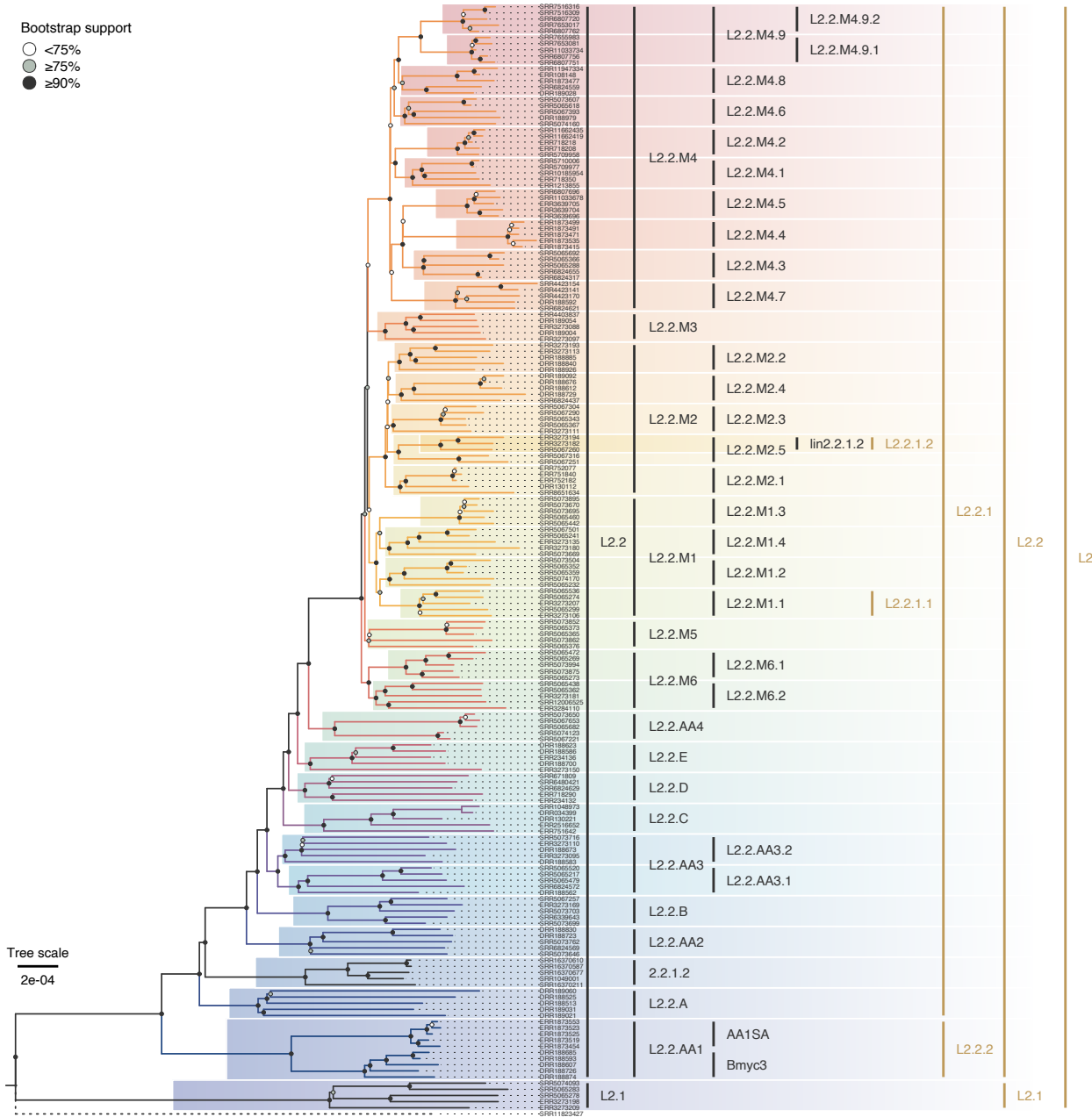

Supplement: Fig S2 — Maximum-likelihood phylogeny of 180 M. tuberculosis lineage 2 isolates. Tree was constructed using 7 919 genome-wide SNPs and rooted on M. tuberculosis H37Rv (SRR11823427 [branch length is omitted]). The exterior vertical bars and names indicate lineage (black - this study, golden - Napier et al.). Lineages are highlighted with background colors. Bootstrap support values are shown as white <75%, grey ≥75% or black ≥90% dots on interior nodes. [file msphere.00169-23-s0002.pdf]

# Bootstrap support

- <75%
- ≥75%
- ≥90%

Tree scale

3e-04

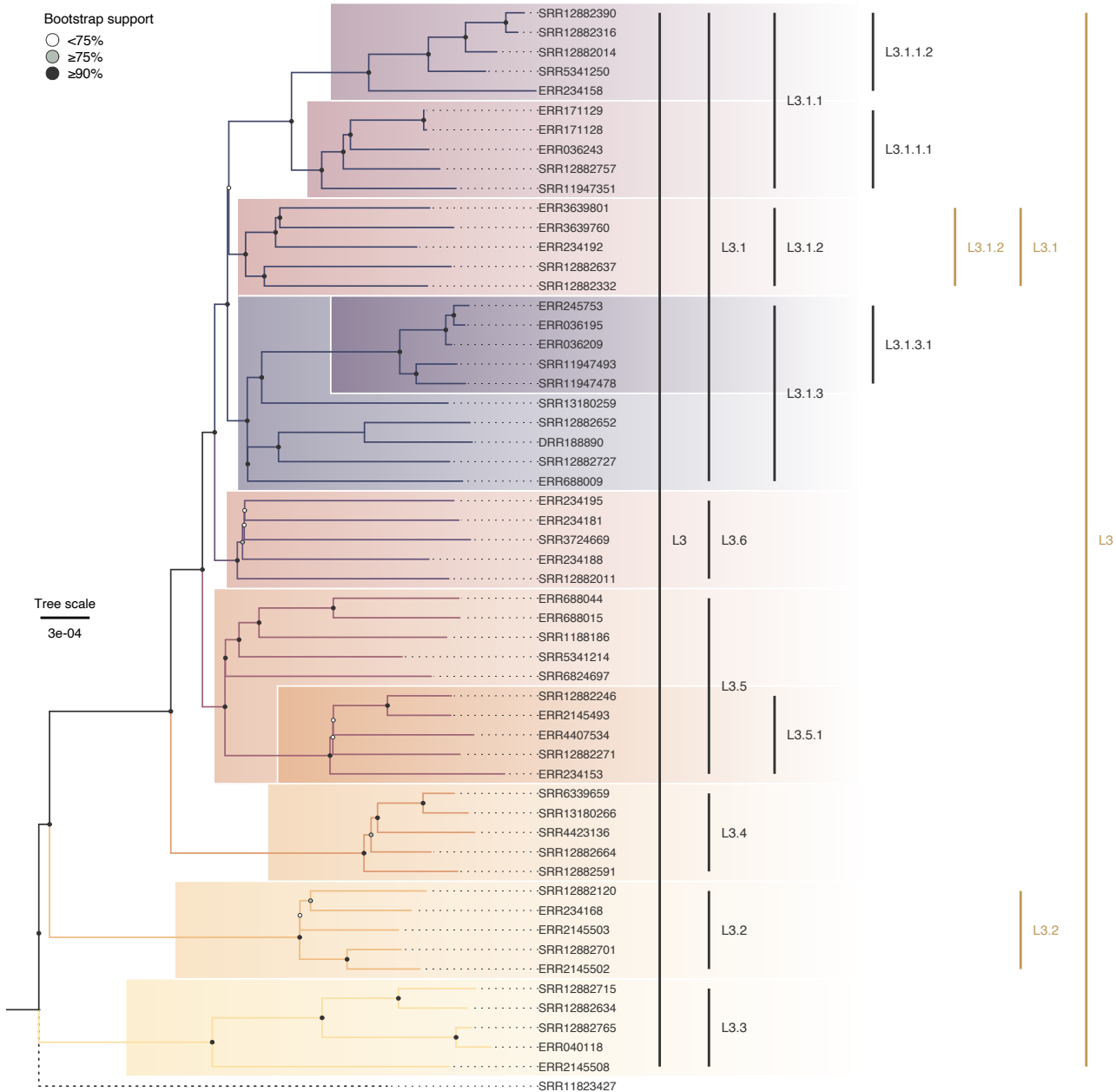

Supplement: Fig S3 — Maximum-likelihood phylogeny of 55 M. tuberculosis lineage 3 isolates. Tree was constructed using 6 047 genome-wide SNPs and rooted on M. tuberculosis H37Rv (SRR11823427 [branch length is omitted]). The exterior vertical bars and names indicate lineage (black - this study, golden - Napier et al.). Lineages are highlighted with background colors. Bootstrap support values are shown as white <75%, grey ≥75% or black ≥90% dots on interior nodes. [file msphere.00169-23-s0003.pdf]

# Bootstrap support

- <75%
- ≥75%
- ≥90%

Tree scale  
5e-05

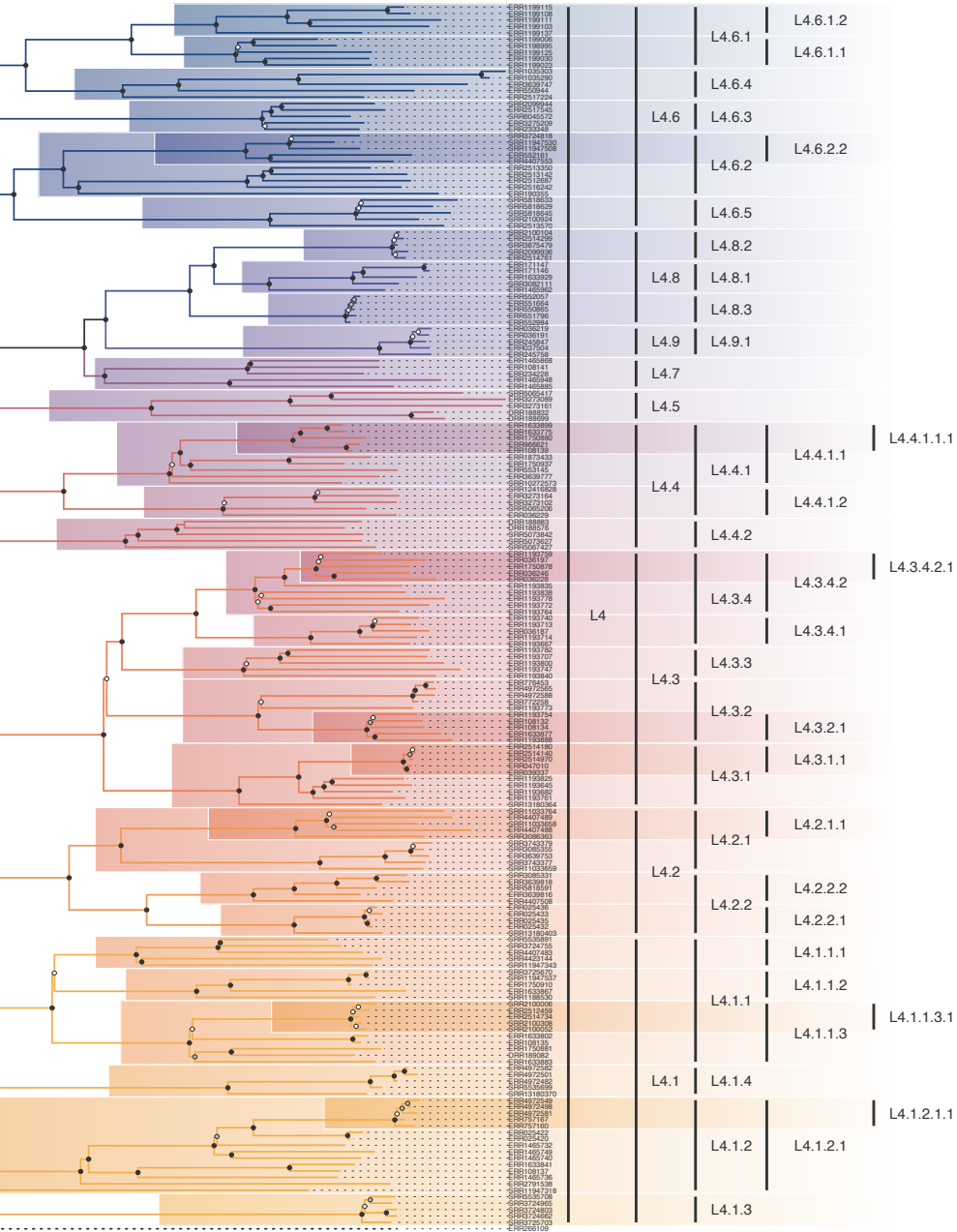

Supplement: Fig S4 — Maximum-likelihood phylogeny of 190 M. tuberculosis lineage 4 isolates. Tree was constructed using 21 471 genome-wide SNPs and rooted on M. canettii (ERR266109 [branch length is omitted]). The exterior vertical bars and names indicate lineage. Lineages are highlighted with background colors. Bootstrap support values are shown as white <75%, grey ≥75% or black ≥90% dots on interior nodes. [file msphere.00169-23-s0004.pdf]

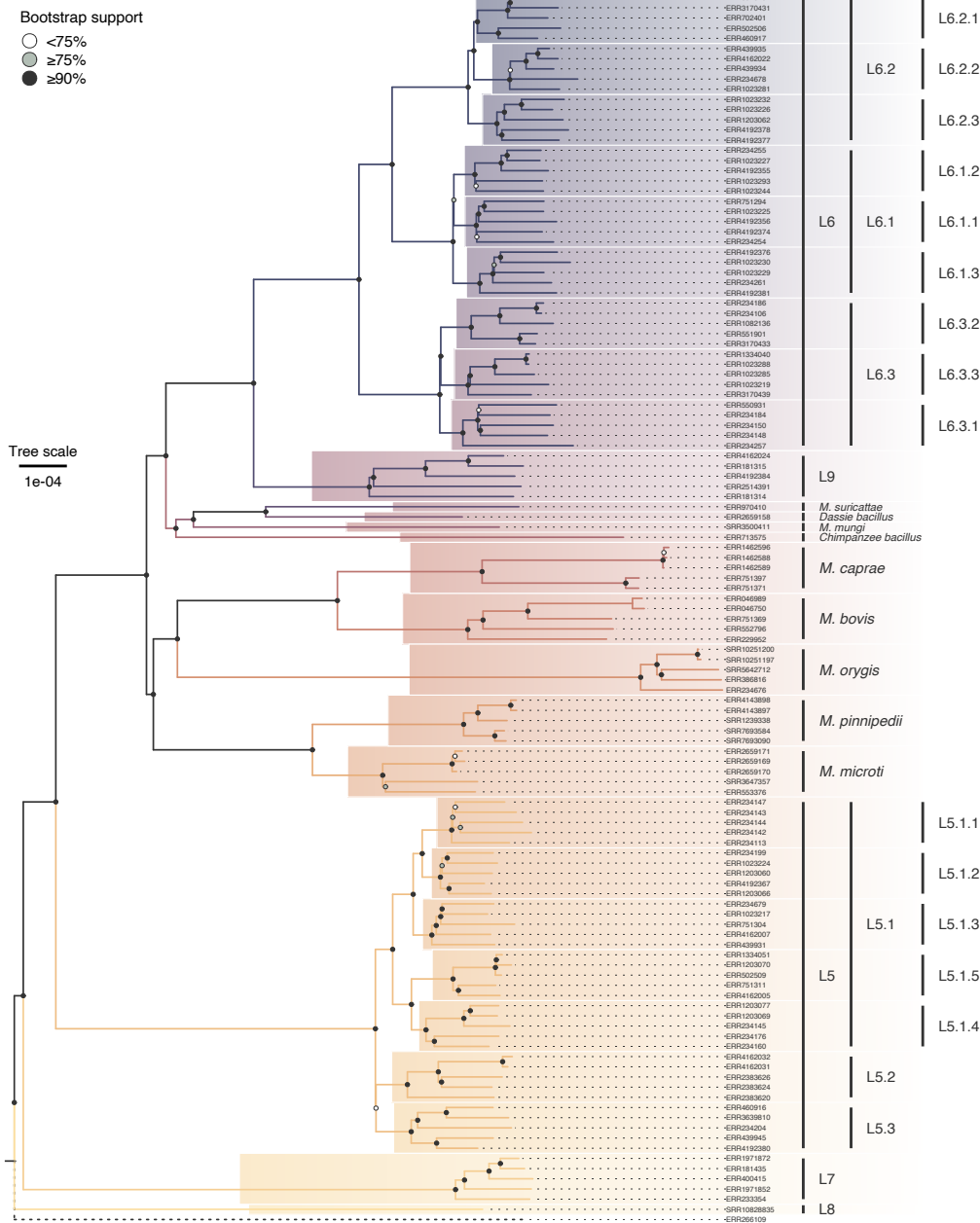

Supplement: Fig S5 — Maximum-likelihood phylogeny of 120 M. tuberculosis, /M. africanum isolates from lineage 5, lineage 6, lineage 7, lineage 8, lineage 9 and animal-adapted species. Tree was constructed using 28 520 genome-wide SNPs and rooted on M. canettii (ERR266109 [branch length is omitted]). The exterior vertical bars and names indicate lineage. Lineages are highlighted with background colors. Bootstrap support values are shown as white <75%, grey ≥75%, or black ≥90% dots on interior nodes. [file msphere.00169-23-s0005.pdf]
