# Supplementary material for: A revised SNP-based barcoding scheme for typing Mycobacterium tuberculosis complex isolates
Source: mSphere. 2023 Jun 14;8(4):e00169-23. doi: 10.1128/msphere.00169-23 (PMC10449489; doi:10.1128/msphere.00169-23)
Supplement: Text S1 — Study design; materials and methods. [file msphere.00169-23-s0006.docx]

# **Supplementary information**

## **Materials and methods**

### **Study design**

### The Napier *et al.* typing scheme and the corresponding barcodes were used as the basis for establishing the MTBC classification (1). Additional classifications were used for lineage 1 (2, 3), lineage 2 (4), lineage 3 (5), lineage 5, lineage 6 (6), and animal-adapted species (7). Additional classifications were compared with the Napier *et al.* typing scheme in terms of names and SNP barcodes, SNPs proposed from the aforementioned studies were used in case of the mismatch. When groups of specific SNPs were presented in corresponding studies for L3, L5 and L6, preference was given towards synonymous mutations in essential genes (8).

### A previously obtained exploratory dataset comprising 9 471 isolates was used for primary screening and differentiation of samples (9). The exploratory dataset was supplemented with samples from the relevant studies for individual lineages, in the absence of samples carrying a particular barcode.

### The final isolates were selected in the following way: a) for each phylogenetic unit, five isolates were selected (when available); b) the average coverage depth was selected at the >80 and >95% reads mapped to the reference genome (If the number of isolates for a specific clade was not sufficient, the rule was omitted); c) preference was given to isolates from different studies. In order to represent the phylogeny and classification as a whole, a level system was used, where the first level corresponds to the main phylogenetic lineage, and the last level corresponds to the final phylogenetic unit. In general, the entire nomenclature was represented by five levels, which were correlated with the primary classification on inferred phylogenetic trees.

### The barcoding system for typing samples was comprised as follows: a) to determine the first two levels of each phylogenetic lineage, two SNPs were used (except L8), for the next levels, one SNP per lineage; b) samples at any of the levels should contain SNPs of the previous level.

### **Dataset**

An exploratory dataset of *Mycobacterium tuberculosis* complex isolates (*n* = 10 297) was retrieved from NCBI database (<https://www.ncbi.nlm.nih.gov/sra>) using SRA Toolkit v3.0.0 (<https://github.com/ncbi/sra-tools>) and parallel-fastq-dump v0.6.7 (<https://github.com/rvalieris/parallel-fastq-dump>). Quality of downloaded FASTQ files was assessed with FastQC v0.11.9 (<https://github.com/s-andrews/FastQC>).

### **Mapping and variant calling**

FASTQ reads were mapped to reference *M. tuberculosis* H37Rv genome (RefSeq accession no. NC_000962.3) using BWA MEM v0.7.17 (10) algorithm. Subsequently, duplicate reads were removed with picard MarkDuplicates v2.27.4 (<https://github.com/broadinstitute/picard>). Mapped reads were sorted by coordinates, converted to BAM format and indexed using SAMtools v1.16.1 (11). Mapping quality was assessed with SAMtools stats and mosdepth v0.3.3 (12). All the following variant calling steps were performed with various tools from GATK4 v4.2 package (13). Variants in each isolate were called with HaplotypeCaller with “-ploidy 1” and “-mbq 20” arguments and imported into GenomicsDB before joint genotyping using GenomicsDBImport. Joint genotyping was performed using GenotypeGVCFs tool “-ploidy 1” argument. Called variants were filtered with VariantFiltration using the following filter expression: “QD < 2.0 || DP < 10 || FS > 60.0 || MQ < 40.0”. Multiallics variants were then split into biallelics, left aligned and trimmed with LeftAlignAndTrimVariants. Only SNPs that passed filtering were selected using SelectVariants and subsequently transformed into tab-delimited format with VariantsToTable. Summary statistics for a final cohort VCF was acquired using stats command from BCFtools v1.15.1 (11). All reports were aggregated with MultiQC v1.10.1 (14).

### **Lineage calling and phylogeny**

Lineages from called SNPs were assigned with custom Python scripts, these scripts are incorporated in TBvar v1.1.3 snakemake workflow available at GitHub (<https://github.com/dbespiatykh/TBvar>). For phylogenies construction, data were subsampled to include *n* = 5 (when possible) isolates per the lowest possible sublineage. Subsampled dataset included all *n* = 169 lineages and sublineages amounting to *n* = 670 isolates: L1 (*n* = 125), L2 (*n* = 180), L3 (*n* = 55), L4 (*n* = 190), and L5-Animal (*n* = 120). SNP alignment was extracted from tab-delimited output from GATK VariantsToTable command. Recombinant regions from the SNP alignment were filtered out using Gubbins v3.2.1 (15). The resulting alignment was cleaned with SNP-sites v2.5.1 (16). Phylogenies were inferred using IQ-TREE 2 v2.2.0.3 (17). Support values were inferred from 1 000 ultrafast bootstrap replicates (UFBoot (18)) with the “-bnni” argument and from 1 000 replicates for SH approximate likelihood ratio test with the “-altr” argument. Best-fit models were determined by ModelFinder (19) with the “-m MFP” argument, models were chosen as follows: TVM+F+ASC+R5 (L1, L2), TVM+F+ASC+R7 (L5-Animal), K3Pu+F+ASC+R4 (L3), and K3Pu+F+ASC+R5 (L4, MTBC). Trees were rooted on *M. tuberculosis* H37Rv [SRR11823427 (L1, L2, L3)] or on *M. canettii* [ERR266109 (L4, L5-Animal, MTBC)]. Phylogenies were visualized with the ggtree v3.2.1 (20), ggplot2 v3.3.6 (<https://ggplot2-book.org/>), ggnewscale v0.4.7 (<https://github.com/eliocamp/ggnewscale>), PNWColors v0.1.0 (<https://github.com/jakelawlor/PNWColors>), and gginnards v0.1.0-1 (<https://github.com/cran/gginnards>) packages for R v4.1.2 (21).

### **Additional tools**

A streamlit (<https://streamlit.io/>) companion web application TB-gen was developed to facilitate MTBC research by providing various functionalities that are helpful for genotyping MTBC isolates. The main functions of TB-gen include a curated list of barcoding SNPs for accurate lineage classification, a reference dataset with detailed information about isolates, and phylogenies to represent the relationships between the aforementioned isolates. “Barcoding SNPs” page includes information on the specific SNPs that correspond to each lineage, the “Phylogeny” page includes a graphical representation of the MTBC lineages and sub-lineages, and the “Reference dataset” page contains detailed information on the reference data source, versions, and quality filtering. Furthermore, TB-gen allows users to barcode tuberculosis lineage from VCF files, this functionality is available on “Genotype lineage” page. The application provides researchers with user-friendly interfaces, allowing for quick and efficient analysis of MTBC samples. When used in combination with existing analytical tools, this web-app is a valuable resource for tuberculosis research, facilitating the accurate and efficient classification of MTBC strains in epidemiological studies.

In addition, we developed a standalone Python command line tool – TbLG, that enables easy lineage classification from a VCF file. The tool employs a set of reference barcoding SNPs identified in this study to classify lineages. To identify lineage of the isolate the tool identifies the SNP patterns in VCF file and compares them with a database of known SNPs that are specific to each lineage. Our tool delivers a dependable and standardized method for lineage classification, which is essential for accurate epidemiological research on tuberculosis.

### **Code availability**

Reproducible variant calling and lineage barcoding workflow TBvar v1.1.5 is implemented in Snakemake v7.18.2 (22) and available at GitHub (<https://github.com/dbespiatykh/TBvar>).

# **References**

1. Napier G, Campino S, Merid Y, Abebe M, Woldeamanuel Y, Aseffa A, Hibberd ML, Phelan J, Clark TG. 2020. Robust barcoding and identification of Mycobacterium tuberculosis lineages for epidemiological and clinical studies. Genome Med 12:1–10.

2. Netikul T, Thawornwattana Y, Mahasirimongkol S, Yanai H, Maung HMW, Chongsuvivatwong V, Palittapongarnpim P. 2022. Whole-genome single nucleotide variant phylogenetic analysis of Mycobacterium tuberculosis Lineage 1 in endemic regions of Asia and Africa. Scientific Reports 2022 12:1 12:1–11.

3. Palittapongarnpim P, Ajawatanawong P, Viratyosin W, Smittipat N, Disratthakit A, Mahasirimongkol S, Yanai H, Yamada N, Nedsuwan S, Imasanguan W, Kantipong P, Chaiyasirinroje B, Wongyai J, Toyo-oka L, Phelan J, Parkhill J, Clark TG, Hibberd ML, Ruengchai W, Palittapongarnpim P, Juthayothin T, Tongsima S, Tokunaga K. 2018. Evidence for Host-Bacterial Co-evolution via Genome Sequence Analysis of 480 Thai Mycobacterium tuberculosis Lineage 1 Isolates. Scientific Reports 2018 8:1 8:1–14.

4. Thawornwattana Y, Mahasirimongkol S, Yanai H, Maung HMW, Cui Z, Chongsuvivatwong V, Palittapongarnpim P. 2021. Revised nomenclature and SNP barcode for Mycobacterium tuberculosis lineage 2. Microb Genom 7:000697.

5. Shuaib YA, Utpatel C, Kohl TA, Barilar I, Diricks M, Ashraf N, Wieler LH, Kerubo G, Mesfin EA, Diallo AB, Al-Hajoj S, Ndung’u P, Fitzgibbon MM, Vaziri F, Sintchenko V, Martinez E, Viegas SO, Zhou Y, Azmy A, Al-Amry K, Godreuil S, Varma-Basil M, Narang A, Ali S, Beckert P, Dreyer V, Kabwe M, Bates M, Hoelscher M, Rachow A, Gori A, Tekwu EM, Sidze LK, Jean-Paul AA, Beng VP, Ntoumi F, Frank M, Diallo AG, Mboup S, Tessema B, Beyene D, Khan SN, Diel R, Supply P, Maurer FP, Hoffmann H, Niemann S, Merker M. 2022. Origin and Global Expansion of Mycobacterium tuberculosis Complex Lineage 3. Genes (Basel) 13:990.

6. Coscolla M, Gagneux S, Menardo F, Loiseau C, Ruiz-Rodriguez P, Borrell S, Otchere ID, Asante-Poku A, Asare P, Sánchez-Busó L, Gehre F, Sanoussi CN, Antonio M, Affolabi D, Fyfe J, Beckert P, Niemann S, Alabi AS, Grobusch MP, Kobbe R, Parkhill J, Beisel C, Fenner L, Böttger EC, Meehan CJ, Harris SR, de Jong BC, Yeboah-Manu D, Brites D. 2021. Phylogenomics of mycobacterium africanum reveals a new lineage and a complex evolutionary history. Microb Genom 7:1–14.

7. Brites D, Loiseau C, Menardo F, Borrell S, Boniotti MB, Warren R, Dippenaar A, Parsons SDC, Beisel C, Behr MA, Fyfe JA, Coscolla M, Gagneux S. 2018. A new phylogenetic framework for the animal-adapted mycobacterium tuberculosis complex. Front Microbiol 9:2820.

8. Dejesus MA, Gerrick ER, Xu W, Park SW, Long JE, Boutte CC, Rubin EJ, Schnappinger D, Ehrt S, Fortune SM, Sassetti CM, Ioerger TR. 2017. Comprehensive essentiality analysis of the Mycobacterium tuberculosis genome via saturating transposon mutagenesis. mBio 8.

9. Bespiatykh D, Bespyatykh J, Mokrousov I, Shitikov E. 2021. A Comprehensive Map of Mycobacterium tuberculosis Complex Regions of Difference. mSphere 6.

10. Li H, Durbin R. 2009. Fast and accurate short read alignment with Burrows–Wheeler transform. Bioinformatics 25:1754–1760.

11. Danecek P, Bonfield JK, Liddle J, Marshall J, Ohan V, Pollard MO, Whitwham A, Keane T, McCarthy SA, Davies RM, Li H. 2021. Twelve years of SAMtools and BCFtools. Gigascience 10:1–4.

12. Pedersen BS, Quinlan AR. 2018. Mosdepth: quick coverage calculation for genomes and exomes. Bioinformatics 34:867–868.

13. Depristo MA, Banks E, Poplin R, Garimella K v., Maguire JR, Hartl C, Philippakis AA, del Angel G, Rivas MA, Hanna M, McKenna A, Fennell TJ, Kernytsky AM, Sivachenko AY, Cibulskis K, Gabriel SB, Altshuler D, Daly MJ. 2011. A framework for variation discovery and genotyping using next-generation DNA sequencing data. Nature Genetics 2011 43:5 43:491–498.

14. Ewels P, Magnusson M, Lundin S, Käller M. 2016. MultiQC: summarize analysis results for multiple tools and samples in a single report. Bioinformatics 32:3047–3048.

15. Croucher NJ, Page AJ, Connor TR, Delaney AJ, Keane JA, Bentley SD, Parkhill J, Harris SR. 2015. Rapid phylogenetic analysis of large samples of recombinant bacterial whole genome sequences using Gubbins. Nucleic Acids Res 43:e15–e15.

16. Page AJ, Taylor B, Delaney AJ, Soares J, Seemann T, Keane JA, Harris SR. 2016. SNP-sites: rapid efficient extraction of SNPs from multi-FASTA alignments. Microb Genom 2:e000056.

17. Minh BQ, Schmidt HA, Chernomor O, Schrempf D, Woodhams MD, von Haeseler A, Lanfear R, Teeling E. 2020. IQ-TREE 2: New Models and Efficient Methods for Phylogenetic Inference in the Genomic Era. Mol Biol Evol 37:1530–1534.

18. Hoang DT, Chernomor O, von Haeseler A, Minh BQ, Vinh LS. 2018. UFBoot2: Improving the Ultrafast Bootstrap Approximation. Mol Biol Evol 35:518–522.

19. Kalyaanamoorthy S, Minh BQ, Wong TKF, von Haeseler A, Jermiin LS. 2017. ModelFinder: fast model selection for accurate phylogenetic estimates. Nature Methods 2017 14:6 14:587–589.

20. Xu S, Li L, Luo X, Chen M, Tang W, Zhan L, Dai Z, Lam TT, Guan Y, Yu G. 2022. Ggtree: A serialized data object for visualization of a phylogenetic tree and annotation data. iMeta 1:e56.

21. R Core Team. 2021. R: A Language and Environment for Statistical Computing. Vienna, Austria.

22. Köster J, Rahmann S. 2012. Snakemake—a scalable bioinformatics workflow engine. Bioinformatics 28:2520–2522.
